# Psychological First Aid by AI: Proof‐of‐Concept and Comparative Performance of ChatGPT‐4 and Gemini in Different Disaster Scenarios

**DOI:** 10.1002/jclp.23808

**Published:** 2025-05-09

**Authors:** Jun Tat Tan, Rick Kye Gan, Carlos Alsua, Mark Peterson, Ricardo Úbeda Sales, Ann Zee Gan, José Antonio Cernuda Martínez, Pedro Arcos González

**Affiliations:** ^1^ Department of Old Age Psychiatry Royal Cornhill Hospital Aberdeen UK; ^2^ Unit for Research in Emergency and Disaster, Area of Preventive Medicine and Public Health, Department of Medicine Universidad de Oviedo Oviedo Asturias Spain; ^3^ McGuire Center for Entrepreneurship University of Arizona Tucson Arizona USA; ^4^ UAI Business School Universidad Adolfo Ibáñez Peñalolén Chile; ^5^ Medicure KB Clinic Kota Bharu Kelantan Malaysia

**Keywords:** AI Chatbot, disaster, frugal innovation, healthcare, psychological first aid

## Abstract

**Objective:**

This study aimed to evaluate the performance and proof‐of‐concept of psychological first aid (PFA) provided by two AI chatbots, ChatGPT‐4 and Gemini.

**Methods:**

A mixed‐method cross‐sectional analysis was conducted using validated PFA scenarios from the Institute for Disaster Mental Health. Five scenarios representing different disaster contexts were selected. Data were collected by prompting both chatbots to perform PFA based on these scenarios. Quantitative performance was assessed using the PFA principles of Look, Listen, and Link, with scores assigned using IFRC's PFA scoring template. Qualitative analysis involved content analysis for AI hallucination, coding responses, and thematic analysis to identify key subthemes and themes.

**Results:**

ChatGPT‐4 outperformed Gemini, achieving an overall score of 90% (CI: 86%–93%) compared to Gemini's 73% (CI: 67%–79%), a statistically significant difference (*p* = 0.01). In the Look domain, ChatGPT‐4 scored higher (*p* = 0.02), while both performed equally in the Listen and Link domain. The content analysis of AI hallucinations reveals that ChatGPT‐4 has a relative frequency of 18.4% (CI: 12%–25%), while Gemini exhibits a relative frequency of 50.0% (CI: 26.6%–71.3%), (*p* < 0.01). Five themes emerged from the qualitative analysis: Look, Listen, Link, Professionalism, Mental Health, and Psychosocial Support.

**Conclusion:**

ChatGPT‐4 demonstrated superior performance in providing PFA compared to Gemini. While AI chatbots show potential as supportive tools for PFA providers, concerns regarding AI hallucinations highlight the need for cautious implementation. Further research is necessary to enhance the reliability and safety of AI‐assisted PFA, particularly by eliminating hallucinations, and to integrate the current advances in voice‐based chatbot functionality.

## 1 Introduction

Psychological first aid (PFA) is a supportive intervention designed to help individuals in the immediate aftermath of a traumatic event. PFA was first introduced during World War II to provide rapid, effective support to those affected by severe stressors, particularly in military contexts (Shultz and Forbes 2014). It is widely recognized that individuals affected by disasters often endure a variety of negative emotions (Bovin and Marx 2011). These emotional, cognitive, and behavioral responses, known *as peritraumatic reactions*, typically manifest as sadness, anger, fear, and apprehension (Massazza et al. 2021). Approximately 22% of people exposed to conflicts or disasters will experience mental health conditions such as depression, anxiety, posttraumatic stress disorder (PTSD), or other serious mental health disorders (World Health Organization 2022). This mental health burden is particularly significant in crisis environments, where the breakdown of community and healthcare systems leaves individuals more vulnerable (Keya et al. 2023). These conditions have long‐lasting effects, not only on the survivors themselves but also on their families and society at large (Bonanno et al. 2010).

In the context of disaster management, PFA is a structured intervention designed to support individuals in the immediate aftermath of a disaster, aiming to alleviate initial distress while fostering long‐term adaptive coping skills (Morgan et al. 2018; World Health Organization 2013). Field evaluations and qualitative studies further reinforce the value of PFA. In community disaster responses, trained helpers have observed that using the Look, Listen, Link approach makes survivors feel heard, supported, and less alone, which in turn helps them start coping better (Everly et al. 2016; Everly and Lating 2021). A qualitative study of PFA implemented after a conflict in Gaza noted that the role of PFA in the acute setting, as delineated by Look, Listen, and Link, is both to provide immediate, humane psychosocial support and to help people begin meeting their basic needs through linking with available services and strengthening their personal coping abilities (Schafer et al. 2015).

Despite variations in PFA models, core principles include promoting safety, engaging in active listening, providing essential information, and connecting individuals with support resources (Hermosilla et al. 2023). Research has demonstrated that PFA effectively reduces symptoms of anxiety (Despeaux et al. 2019) and PTSD (Cain et al. 2010), while also fostering mental well‐being (Blake et al. 2020) and emotional security (de Freitas Girardi et al. 2020). However, the evaluation of PFA's efficacy is hindered by inconsistencies in intervention components, methodological limitations in outcome assessments, and a high risk of bias across existing studies (Hermosilla et al. 2023). Furthermore, a notable disparity persists between the widespread endorsement of PFA and the limited empirical evidence derived from clinical trials and robust outcome data (Wang et al. 2024).

The timing of interventions following exposure to a traumatic event, often referred to as the “golden hours,” is critical as it directly influences their effectiveness in preventing conditions like PTSD (Carmi et al. 2016). Nevertheless, the shortage of PFA providers is a well‐documented issue (Philips 2023), particularly in disaster‐stricken countries and in the aftermath of crises, where the demand for psychological support far exceeds the available resources (Gaiser et al. 2023; Goto et al. 2022; Abed Alah 2024). In response to the shortage of mental health providers, a digital self‐help tool in the form of a chatbot was deployed to promote mental well‐being among Ukrainians affected by the war (Frankova et al. 2022; Lahutina et al. 2024). Remarkably, within just 3 months of the war's onset, from March 2022 to the end of May 2022, the chatbot had engaged with 50,000 users offering mental health support and PFA (Lahutina et al. 2024).

Open‐source AI chatbot models like ChatGPT, which became available in November 2022, followed by Google Bard, later renamed Gemini, represent a promising development in this field. By September 2024, both AI chatbots have seen more than 45 million mobile app downloads (Global ChatGPT and Gemini app downloads 2024). These accessible and ubiquitous AI chatbots are envisioned to play a critical role in the future of PFA (Aditama et al. 2023; Taiwo and Al‐Bander 2025), particularly during disasters when demand for mental health support overwhelms available resources.

This study aims to evaluate the performance and proof‐of‐concept of PFA provided by two AI chatbots, ChatGPT‐4 and Gemini. The focus will be on assessing their performance, analyzing the content of their responses, and examining the outcomes of their interactions.

## 2 Methods

This study utilizes mixed‐method cross‐sectional analysis to assess the performance of ChatGPT‐4 and Gemini in performing PFA in different disaster scenarios. Data was collected from March 22 to 25, 2024 and analyzed by JTT, a practicing psychiatrist, and RKG, a disaster public health physicians and researcher, both trained and experienced in providing PFA. No ethical considerations were needed since all data used were open‐source and secondary sources.

### 2.1 Materials

With written permission, the study employed validated PFA scenarios developed by the Institute for Disaster Mental Health (IDMH). The PFA scenarios were developed in 2016, together with the Psychological First Aid Demonstration Project Preparedness and Emergency Response Learning Center (PERLC), aiming to train and access the PFA participant (Preparedness and Emergency Response Learning Center (PERLC) 2016).

For the study, five questions were selected, encompassing two natural hazard‐related scenarios, one mass shooting scenario, one technological disaster scenario, and one pandemic scenario, ensuring a balanced representation of PFA applications. Each question has subquestions ranging from 9 to 12. Full questions are available in Supporting Information S1: Appendix I. The PFA scenarios included are:

Question 1 Hospital‐based Family Assistance Center, assisting a family member following a worksite explosion.

Question 2 Emergency shelter following a tornado, assisting an evacuee.

Question 3 Disaster recovery center following a major flood after a hurricane, providing PFA to a first responder.

Question 4 Avian flu pandemic point‐of‐distribution (POD), assisting mother of two young children.

Question 5 School mass shooting, assisting the family of a child survivor.

### 2.2 Data Collection

JTT and RKG collected data, testing ChatGPT‐4 and Gemini's ability to perform PFA based on the given disaster scenarios, using the prompts “I would like you to perform psychological first aid on a client based on a scenario as a PFA volunteer.” The PFA scenarios were then presented to the chatbot individually, and the responses from both AI chatbots were recorded in Microsoft Word for detailed analysis.

### 2.3 Data Analysis

The performance of the PFA provided by ChatGPT‐4 and Gemini was evaluated using the PFA training feedback form (IFRC 2018) designed by the Psychosocial Centre of the International Federation of Red Cross and Red Crescent Society (IFRC). The assessment forms are structured around the three core principles and actions of PFA: Look, Listen, and Link. The marking of the performance in these categories was scored based on the degree of completion, with scores assigned as follows: 2 for “Done well,” 1 for “Done adequately,” and 0 for “Not done,” as shown in Supporting Information S2: Appendix II. The scores were given independently by JTT and RKG. The assigned scores were then reviewed by all the other co‐authors for verification. During this verification process, the chatbot identity was blinded, and the verification was conducted objectively. There were no disagreements among the co‐authors regarding the assigned scores. Given the ordinal nature of the scores and the small sample size, the Mann–Whitney *U* Test was chosen to determine the statistical significance of the differences between the performance of ChatGPT‐4 and Gemini across the domains, with a 95% confidence interval established for the mean percentage comparisons.

The PFA responses provided by the AI chatbots were subjected to qualitative analysis. Initially, content analysis was performed to identify AI hallucinations. This involved a systematic examination of the responses to detect any instances where the AI chatbots may have provided inaccurate or inappropriate information. Frequencies of AI hallucination were recorded, and relative frequencies were calculated. A proportions *Z*‐test was then conducted to compare the outcomes, with the significance level set at *p* = 0.05. The responses were then coded, which was then followed by both deductive and inductive thematic analyses. The deductive thematic analysis was structured around the three foundational PFA principles: “Look, Listen, and Link.” Meanwhile, the inductive analysis aimed to identify additional emergent themes from the outputs of the AI chatbots.

## 3 Results

**Figure 1:**
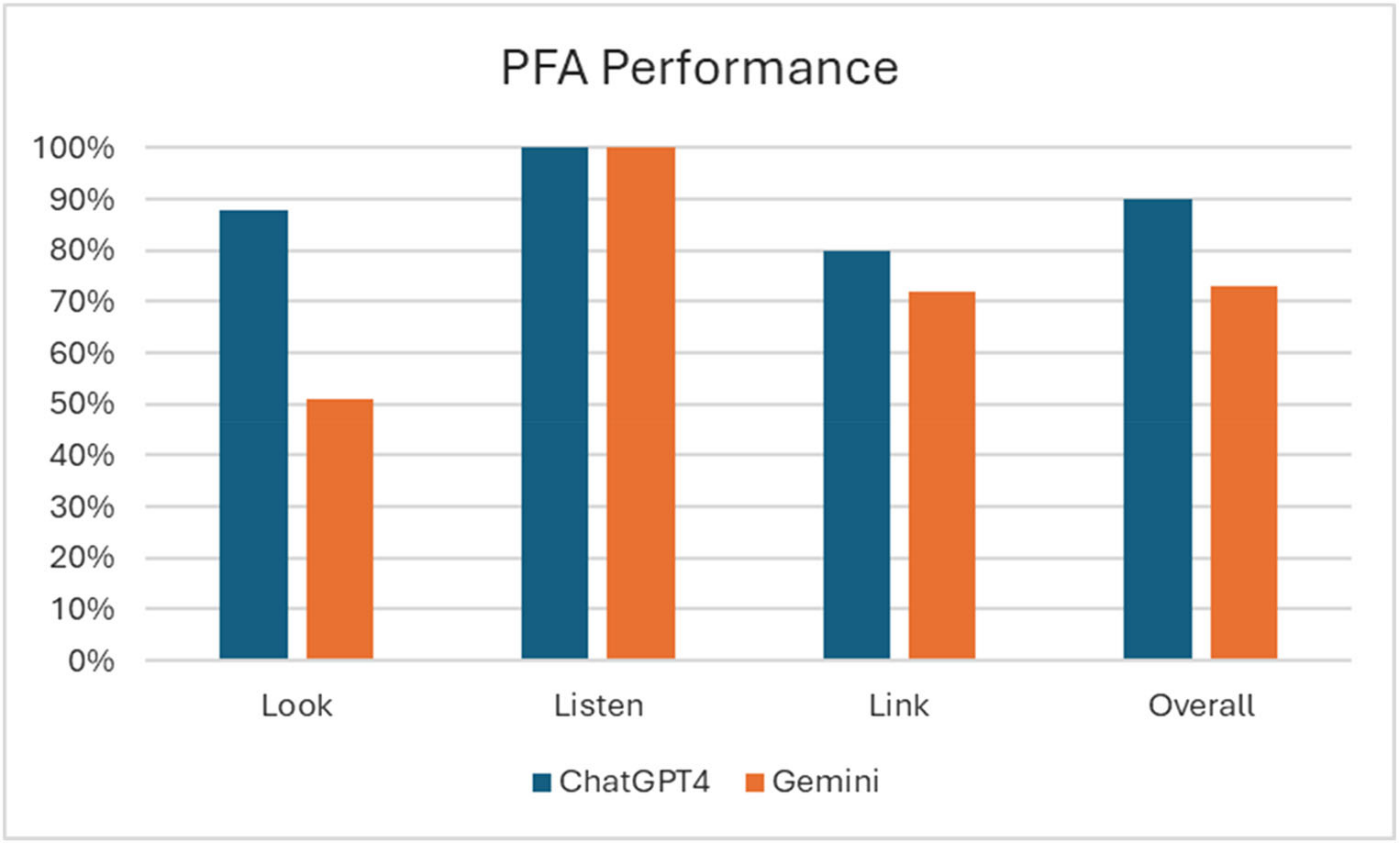
Average scores of ChatGPT‐4 and gemini across all PFA domains.

The results for the Look domain show that ChatGPT‐4 scored significantly higher than Gemini. ChatGPT‐4 achieved an overall score of 10.6/12, 88% (CI: 76%–100%), while Gemini scored 6.2/12 51% (CI: 42%–60%). This difference was statistically significant (*p *= 0.02). ChatGPT‐4 and Gemini performed equally well in the Listen domain, each achieving a perfect score of 10/10, 100% (CI ‐ ‐) across all scenarios. As a result, there was no statistically significant difference between the two AI models in this domain (*p *= 0.9). For the Link domain, ChatGPT‐4 once again outperformed Gemini, scoring 6.4/8, 80% (CI: 71%–89%), compared to Gemini's score of 5.8/8, 72% (CI: 52–92%). However, the Link domain difference was statistically insignificant (*p *= 0.34). Regarding the overall score, ChatGPT‐4 obtained a total score of 27/30, 90% (CI: 86%–93%), while Gemini scored 22/30, 73% (CI: 67%–79%). This overall difference was statistically significant (*p *= 0.01), as shown in Tables 1 and 2 and Figure 1.

**Table 1:** Comparative scores of ChatGPT‐4 and gemini across all PFA domains.

PFA domain	Look						Listen						Link						Overall					
Questions	1	2	3	4	5	Total	1	2	3	4	5	/X	1	2	3	4	5	/X	1	2	3	4	5	/X
ChatGPT‐4 score	10/12	9/12	11/12	12/12	11/12	10.6/12	10/10	10/10	10/10	10/10	10/10	10/10	7/8	7/8	6/8	6/8	6/8	6.4/8	27/30	26/30	27/30	28/30	27/30	27/30
	83%	75%	91%	100%	91%	88%	100%	100%	100%	100%	100%	100%	88%	88%	75%	75%	75%	80%	90%	86%	90%	93%	90%	90%
Gemini score	5/12	6/12	7/12	7/12	6/12	6.2/12	10/10	10/10	10/10	10/10	10/10	10/10	7/8	4/8	7/8	6/8	5/8	5.8/8	22/30	20/30	24/30	23/30	21/30	22/30
	41%	50%	58%	58%	50%	51%	100%	100%	100%	100%	100%	100%	87%	50%	87%	75%	62%	72%	73%	67%	80%	76%	70%	73%

**Table 2:** Comparative analysis of scores achieved by ChatGPT‐4 and Gemini across all PFA domains.

Overall	ChatGPT‐4	Gemini	*p* value
Look	88% (CI: 76%–100%)	51% (CI: 42%–60%)	*p* = 0.02
Listen	100% (CI: ‐ ‐)	100% (CI: ‐ ‐)	*p* = 0.9
Link	80% (CI: 71%–89%)	72% (CI: 52%–92%)	*p* = 0.34
Overall	90% (CI: 86%–93%)	73% (CI: 67%–79%)	*p* = 0.01

### 3.1 Content Analysis for AI Hallucination

AI hallucination refers to the generation of content that is not grounded in accurate or existing data but is produced by a machine learning model's extrapolation or creative interpretation of its training data (Hatem et al. 2023). We identified several instances of AI hallucination in our analysis of ChatGPT‐4 and Gemini's responses to PFA scenarios. These included irrelevant or unprompted responses, such as providing PFA guidelines or principles without being asked and engaging in role‐play through scripted dialog. Additionally, we observed fabrication‐type hallucinations, where the AI‐generated fictitious character names provided context unrelated to the prompt or assumed incorrect locations, such as offering support services exclusive to the United States, as shown in Table 3. The comparative analysis of AI hallucination frequency indicates that in ChatGPT‐4's responses, hallucinations occurred nine times out of 49 questions, corresponding to a relative frequency of 18.4% (CI: 12%–25%). In contrast, Gemini exhibited hallucinations 24 times out of 49 questions, resulting in a relative frequency of 50% (CI: 26.6%–71.3%), *p *= 0.0013, as shown in Tables 4 and 5.

**Table 3:** Frequency of AI hallucinations identified in ChatGPT‐4(C) and gemini(G)‘s PFA responses through content analysis.

AI hallucinations		Q1		Q2		Q3		Q4		Q5		Total
		C	G	C	G	C	G	C	G	C	G	
Irrelevance	Unprompted answers	1	0	1	0	2	0	2	0	2	0	C: 8 G: 0
	Script writing	0	1	1	1	0	3	0	1	0	1	C: 1 G: 7
Fabrication	Character	0	5	0	3	0	0	0	0	0	2	C: 0 G: 10
	Location	0	1	0	1	0	2	0	1	0	1	C: 0 G: 6
	Context	0	0	0	0	0	0	0	0	0	1	C: 0 G: 1
Total		1	7	2	5	2	5	2	2	2	5	C: 9 G: 24

**Table 4:** Comparative description of the relative frequency of AI hallucinations in ChatGPT‐4 and gemini.

Question	ChatGPT‐4	%	Gemini	%
1	Irrelevance:1/10	10.0	Irrelevance:1/10	10.0
	Fabrication:0/10	0	Fabrication:6/10	60.0
2	Irrelevance:2/9	22.2	Irrelevance:1/9	11.1
	Fabrication:0/9	0	Fabrication:4/9	44.4
3	Irrelevance:2/9	22.2	Irrelevance:3/9	33.3
	Fabrication:0/9	0	Fabrication:2/9	22.2
4	Irrelevance:2/9	22.2	Irrelevance:1/9	11.1
	Fabrication:0/9	0	Fabrication:1/9	11.1
5	Irrelevance:2/12	16.7	Irrelevance:1/12	8.3
	Fabrication:0/12	0	Fabrication:4/12	33.3
Total	9/49	18.4	24/49	50.0

**Table 5:** Comparative analysis of overall AI hallucinations in ChatGPT‐4 and gemini.

	ChatGPT‐4	Gemini	*p* value
Relative frequencies of AI hallucination	18.4% (CI: 12%–25%)	50% (CI: 26.6%–71.3%)	0.0013

### 3.2 Qualitative Analysis Overview

In each of the five PFA scenarios, between 9 and 12 subprompts were administered, yielding a total of 49 distinct prompts per AI chatbot. On average, each response comprised approximately 500–1700 words, resulting in an overall corpus of 130,000 words analyzed. Our qualitative analysis identified several salient themes in the responses from both AI models, which correspond to the core PFA principles of Look, Listen, and Link, in addition to themes pertaining to Professionalism and Mental Health and Psychosocial Support.

### 3.3 Qualitative Analysis for ChatGPT4's PFA Response

The qualitative analysis of ChatGPT‐4's responses to the PFA scenarios identified several key themes and subthemes that align with the core principles of PFA: Look, Listen, and Link, as well as additional themes related to professionalism and mental health support, as shown in Table 6.

**Table 6:** Emergent themes and subthemes from ChatGPT‐4's responses in PFA scenarios.

ChatGPT4 themes	ChatGPT4 subtheme
1. Look	Assessment of needs and state
2. Listen	Accepting and acknowledging feelings
	Empathic responses and reassurance
	Transparent and honest communication
3. Link	Providing and managing information
	Immediate practical assistance
	Offering support and backup
	Linking resources and services
4. Professionalism	Respect and confidentiality
	Seek guidance and advice from professionals
5. Mental health and psychosocial support	Mental and emotional preparation
	Encouragement and support for self‐care
	Distraction from stress
	Establishing and maintaining routines
	Calming and relaxation techniques
	Planning for the future

1. *Look*

Within the Look domain, ChatGPT‐4 effectively evaluated the essential needs and emotional states of individuals in crisis. Its responses focused on gathering key information to identify immediate needs. For instance, in Question 1.2, a young woman at the hospital was asked:

…Can you tell me a bit more about what you've heard so far, and how you're feeling right now?

When a first responder expressed frustration in Question 3.6, ChatGPT‐4 further inquired:

Have you been able to discuss your feelings and experiences with someone you trust, like a family member, … or a mental health professional?

In Question 5.2, regarding a school shooting, it assessed a daughter's wellbeing by asking:

If you're comfortable, can you share a bit more about how your daughter has been since the incident? Has she been sleeping, eating or behaving differently?

2. *Listen*

Within the Listen domain, ChatGPT‐4 consistently acknowledged and accepted users' feelings through empathetic and reassuring responses, fostering active and transparent communication. For example, in Question 1.2, a young woman was met with:

I understand this is a very distressing time for you. We're here to help you…

In a tornado shelter scenario (Question 2.2), ChatGPT‐4 validated an 18‐year‐old's emotions by stating:

…I understand that you've been through an extremely challenging experience with the tornado. It's okay to feel overwhelmed or upset about what has happened. I'm here to listen and support you…

When a first responder expressed self‐blame in Question 3.4, ChatGPT‐4 responded:

It's clear that you're deeply committed to your work and the well‐being of the people you're trying to help. Feeling overwhelmed in such a demanding and high‐pressure situation is completely understandable.

In an avian flu distribution scenario (Question 4.6), it reassured a concerned mother:

It sounds like you're facing a lot of stress right now. This is certainly a challenging situation, and your feelings of worry and frustration are completely understandable.

Lastly, in Question 1.9, following a workplace explosion, it addressed a wife's anxiety by stating:

I understand why you might feel this way, especially in a situation filled with so much uncertainty and stress. However, I assure you, I don't have access to any information about your husband's condition or the situation at the factory… Remember, you're not alone in this. I'm here to help in any way I can…

and further emphasized transparency with:

…We are getting information as quickly as possible. As soon as we have any news about your husband, we will let you know immediately.

3. *Link*

In In the “Link” theme, ChatGPT‐4's responses concentrated on delivering practical guidance and connecting individuals to appropriate resources. For instance, in Question 5.10, which involved assisting the family of a child survivor of a school shooting, ChatGPT‐4 responded:

…Considering homeschooling or moving are significant decisions that reflect your desire to create a safe environment for her… Here are some points to consider while you navigate these thoughts:

School's Response: Understand how the school is responding to this tragedy. Are they implementing new safety measures? What kind of support (counseling, security enhancements) are they offering to students and families?

ChatGPT‐4 emphasized the importance of staying informed about the school's response in the decision‐making process. In the immediate practical assistance subtheme, when an 18‐year‐old in a shelter expressed concerns about a missing pet in Question 2.2, it responded with:

…Let's see if there are any local services here, like a message board or a communication center set up for survivors, where you can leave a message for your family. We can also ask around if anyone has information on missing pets, which might help in finding your dog

Furthermore, addressing physical discomfort in Question 2.8, ChatGPT‐4 advised:

Is it alright if I ask someone from the medical team to come and check on you? It's important to ensure that you're physically okay. In the meantime, try to take slow, deep breaths, and let's find you a comfortable place to sit or lie down …

In the offering support and backup subtheme, when assisting a mother at an Avian flu pandemic Point‐of‐distribution in Question 4.5, ChatGPT‐4 provided detailed steps:

In a situation like this, it's important to stay calm and take practical steps to protect yourself and your children: 1. Maintain Distance: If possible, discreetly create more space between yourself and the person who is coughing…. Practice Good Hygiene: Use hand sanitizer if you have it, and avoid touching your face. This can help reduce the risk of infection. 3. Wearing Masks: Make sure you and your children are properly wearing masks, as they can provide a barrier against respiratory droplets. 4. Stay Calm for Your Children: Children often look to adults for cues on how to react in stressful situations. Try to stay calm and reassuring, as this can help your children feel more secure…

Additionally, it suggested:

…If the situation feels untenable, assess if there are other options for waiting, like stepping out of line momentarily while maintaining your place, or asking staff if there's a way to wait in a less crowded area…

Finally, in linking resources and services, when a young woman in Question 1.7 sought support after a major explosion, ChatGPT‐4 connected her to local assistance by stating:

Hospitals often have social workers or patient advocates who can provide support during times like these. They can also connect you with local resources or support groups…Sometimes, local community centers or religious organizations offer support, especially in times of crisis…

Overall, ChatGPT‐4's multipronged approach under the Link theme reflects its commitment to providing actionable information, practical assistance, and connecting users to essential services in crisis situations.

4. *Professionalism*

Within the Professionalism theme, ChatGPT‐4 consistently maintained respect and confidentiality. In Question 3.8, a first responder at a hurricane disaster recovery center remarked:

Sorry, ignore me, I'm just tired. Please don't tell my supervisor I said any of this, okay? I don't want to lose this job.

In response, ChatGPT‐4 affirmed the confidentiality of the conversation by stating:

…Your feelings are valid, and it's important to have a space where you can share them safely. Please know that our conversation is confidential, and I won't share any details with your supervisor or anyone else…

Additionally, when addressing concerns about the recovery of an 8‐year‐old child who had witnessed a traumatic event, ChatGPT‐4 underscored the importance of seeking professional guidance. In that scenario, upon the parent's inquiry, “Will she ever recover from this?”

it responded:

Recovery from such a traumatic event is certainly possible…This includes support from family, friends, and possibly professional therapists who specialize in trauma and children… A mental health professional experienced in trauma can provide invaluable support…

These examples illustrate ChatGPT‐4's commitment to upholding professional standards by ensuring confidentiality and by advocating for the involvement of specialized professionals when addressing complex emotional and psychological issues.

5. *Mental Health and Psychosocial Support*

The theme of Mental Health and Psychosocial Support encompassed several subthemes. For instance, under Mental and Emotional Preparation (Question 1.10), when the wife of a missing husband asked, “Do you have any other advise for me?”

ChatGPT‐4 advised:

…While hoping for the best, it can be helpful to mentally prepare for different scenarios. This doesn't mean expecting the worst, but rather equipping yourself to handle a range of possibilities…

In the Encouragement and Support for Self‐Care subtheme (Question 3.7), a first responder expressed:“I'm really not up for doing this anymore. I'm just not able to cope.” ChatGPT‐4 responded:

… Give yourself time to reflect on your experience and to recover from the stress. It's important to engage in self‐care practices and activities that bring you comfort and joy.

For Distraction from Stress (Question 4.2), when a mother waiting in line for a vaccine expressed anxiety, ChatGPT‐4 recommended:

…Share simple coping strategies she can use while waiting, like deep breathing exercises or engaging her children in a light conversation or game to distract them and herself from the stress…

In the subtheme Establishing and Maintaining Routines (Question 5.4), upon hearing,“She's barely said a word since this happened, but she won't let me out of her sight,” ChatGPT‐4 suggested:

…Maintain a routine as much as possible to provide her with a sense of normalcy and stability… Your daughter's gradual return to her usual self is possible with the right support and care.

Regarding Calming and Relaxation Techniques (Question 1.5), when a wife stated, “He's not picking up his phone or answering my texts and he always gets back to me right away. I know something is terribly wrong.” ChatGPT‐4 offered:

…I'm here to support you during this waiting period. If there's anything specific that might help you feel a bit more at ease while we wait for information—maybe talking about something else, focusing on breathing exercises, or finding a quiet space…

Finally, in the Planning for the Future subtheme (Questions 3.6 and 3.7), a first responder was encouraged with:

If you decide to leave, think about what you would like to do next. Do you have other plans for your retirement that you'd like to pursue?… Think about what you'd like to do next. This could be a time to explore other interests or activities that are less stressful and more aligned with your current needs…

Overall, these responses demonstrate a comprehensive approach to psychosocial support, where ChatGPT‐4 addresses immediate emotional needs while also promoting long‐term strategies for coping and recovery.

### 3.4 Qualitative Analysis of Gemini's PFA Response

In the analysis of Gemini's responses to PFA scenarios, five primary themes were identified: Look, Listen, Link, Mental Health, and Psychosocial Support. Each theme encompasses several subthemes that capture the specific aspects of the AI's engagement across various disaster scenarios, as shown in Table 7.

**Table 7:** Emergent themes and subthemes from gemini's responses in PFA scenarios.

Gemini themes	Gemini subtheme
1. Look	Assessment of incident
2. Listen	Accepting and acknowledging feelings
	Empathic responses and reassurance
	Transparent and honest communication
3. Link	Providing and managing information
	Immediate practical assistance
	Linking realistic resources and services
4. Professionalism	Ensure confidentiality
	Seek guidance and advice from professionals
5. Mental health and psychosocial support	Mental and emotional preparation
	Adaptation and flexibility
	Physical and mental health management
	Maintaining routine and normalcy
	Calming and relaxation techniques
	Creating a safe environment

1. *Look:*

This theme illustrates Gemini's capacity to assess situational context and the specific characteristics of incidents. The subtheme, Assessment of Incidents, demonstrates Gemini's approach to evaluating clients' needs by considering both the event's nature and its potential impact. This is exemplified in Question 2.4 (Emergency Shelter following a tornado), where an evacuee expressed concern: “They(her family) must be freaking out about how I am. I bet they're watching the news non‐stop, or maybe they're trying to get home soon. But I heard the airport's closed indefinitely so I don't know how they'll be able to come back.”

Gemini responded by shifting the focus to immediate needs:

…While you can't control the phone lines, the airport closure, or the damage to your house, you can focus on taking care of yourself right now. Is there anything you need ‐ a blanket, some water, a phone charger?… Is there anything else you'd like to talk about?

2. *Listen*

Gemini's PFA response thematic analysis reveals a strong alignment with the Listen domain through empathetic engagement and clear communication. Under the subtheme Accepting and Acknowledging Feelings, Gemini validates users' emotions. For instance, in Question 1.3, when a family member stated, “The police told me to come here but now no one will tell me anything about my husband. Why won't they let me see him?” Gemini responded:

It sounds absolutely terrifying, waiting here with all this uncertainty swirling around you. No wonder you're feeling so scared and frustrated. It's completely understandable that you'd want to see Mark (AI hallucinated name) right away, especially after such a traumatic event.

In the subtheme Empathic Responses and Reassurance, Gemini addresses distress by offering comfort. As demonstrated in Question 3.4, when a FEMA first responder remarked, “I'm doing my best but there are just so many of them, and the paperwork for each case takes freaking forever to complete. I feel like it's my fault. If only I could work faster.” Gemini replied:

It sounds like you're feeling overwhelmed by the workload and the frustration of the situation. It's completely understandable…It's not your fault that there are so many people waiting, or that the paperwork takes time…You're doing the best you can in a very difficult situation.

Lastly, in the subtheme Transparent and Honest Communication, Gemini ensures clarity and openness. For example, in Question 1.9, after a family member expressed frustration by stating, “You know what's going on, don't you? I think you know something you're not telling me.” Gemini maintained transparency by stating:

…I can assure you that I don't have any private information about his specific situation… Hospitals are very careful about protecting patient privacy, and I wouldn't be able to access any medical records even if I wanted to. My role here is to offer you support and help you navigate this difficult time…

Overall, these examples illustrate Gemini's commitment to empathetic, clear, and supportive communication within the Listen domain, effectively addressing the emotional needs of individuals in crisis.

3. *Link*

The thematic analysis of Gemini's responses reveals a strong emphasis on linking practical assistance with appropriate resources. In the subtheme Providing and Managing Information, Gemini tailored its responses to the specific context. For example, in Question 4.4 at an Avian flu pandemic Point‐Of‐Distribution, a concerned mother asked: “I heard that since they had to rush the production of the vaccine, it's really unsafe and tons of people are getting sicker from it than if they got the actual flu. Is that true?” Gemini responded by providing contextually appropriate information:

It's understandable to be concerned about a new vaccine, especially with all the information circulating online. Here's what I can tell you:

Safety Testing: Even though the vaccine was produced quickly, it still went through rigorous safety testing before being approved. Safety is always the top priority for health officials.

Monitoring: Just like any vaccine, this one will be closely monitored for any side effects. The risk of serious side effects is extremely low.

The Flu vs. Vaccine: The potential side effects of the vaccine are far milder than getting the actual avian flu, which can be very dangerous for young children.

Under the subtheme Immediate Practical Assistance, Gemini provided actionable guidance during crises. In Question 3.4, after a FEMA first responder expressed exhaustion by stating: “I'm doing my best but there are just so many of them, and the paperwork for each case takes freaking forever to complete. I feel like it's my fault. If only I could work faster.” Gemini advised:

…Have you had a chance to take a break at all today? Sometimes a few minutes to step away, grab some water, or take a deep breath can make a big difference when you're feeling overloaded. There's no shame in needing a moment to recharge…

Finally, the subtheme Linking Realistic Resources and Services is illustrated in Question 5.11. When a parent of an 8‐year‐old child survivor of a school mass shooting stated: “Ugh, I haven't reached out to Amy's parents yet and I feel terrible about it. I just don't know what to say to them. I mean, I still have my daughter and they don't, so what can I possibly say to them?” Gemini responded by linking the parent to available support resources:

…Here are some resources that might be helpful for you when reaching out:

The Dougy Center: https://www.dougy.org/program-finder offers resources for families coping with grief, including tips on talking to children about death.

The Compassionate Friends: https://www.compassionatefriends.org/ offers grief support groups for parents who have lost a child.

Overall, Gemini's responses under the Link theme demonstrate a multifaceted approach that integrates context‐specific information, immediate practical assistance, and realistic resource connections to effectively support individuals in crisis.

4. *Professionalism*

Gemini's responses further illustrate its commitment to professionalism by emphasizing both the protection of personal information and the encouragement of seeking expert advice. In the subtheme Ensuring Confidentiality, Gemini respects user privacy. For example, in Question 3.8 at a disaster recovery center following a hurricane, after a first responder stated, “Sorry, ignore me, I'm just tired. Please don't tell my supervisor I said any of this, okay? I don't want to lose this job.” Gemini responded,

Absolutely, everything you've shared stays confidential between us.

This response highlights Gemini's careful handling of sensitive disclosures.

In addition, the subtheme Seek Guidance and Advice from Professionals is demonstrated in Question 4.8, where a mother at an Avian flu pandemic Point‐Of‐Distribution expressed concern by stating, “I'm feeling kind of short of breath. I think I may be getting sick already….” Recognizing its limitations, Gemini advised,

… Seek Medical Attention: Since it's important to rule out the flu and other possibilities, it would be best to speak to a medical professional. This vaccination site likely has medical personnel on hand. Would you like me to help you find someone who can check you out?

Overall, these examples underscore Gemini's dual focus on maintaining confidentiality and appropriately directing users to professional resources, thereby reinforcing its adherence to professional standards in crisis response.

5. *Mental Health and Psychosocial Support:*

Gemini's responses reveal a comprehensive approach to Mental Health and Psychosocial Support, articulated through several interrelated subthemes. In Question 1.8, for example, a wife at a Hospital‐based Family Assistance Center following a worksite explosion expresses her deep‐seated concerns: “We've been trying to get pregnant and maybe I already am. What if I am and he's really badly hurt or even…. But he's not. He's going to be okay for our baby. Right? I should call his mother, I know, but she's just going to blame me for this, like she does for everything. Do I have to call her?” Gemini's response methodically guides her through mental and emotional preparation by advising,

…Here's what we can tackle one step at a time: …Right now, your focus and energy need to be on yourself and your own well‐being. However, if you think she might have information about Mark or could offer some kind of support (even if emotional), then a call might be helpful… It's important to acknowledge your fear, but it's also okay to hold onto hope… Focusing solely on the worst‐case scenario won't help you or the baby you might be carrying…

In a similar vein, the subtheme of Adaptation and Flexibility is evident in Question 3.7, where a distressed first responder at a disaster recovery center after a hurricane declares, “I'm really not up for doing this anymore. I'm just not able to cope.” Gemini responds by promoting adaptability through concrete suggestions, stating,

…Here are some next steps to consider: Talking to your supervisor: Let them know that the stress of the job is becoming overwhelming for you, and that you're considering quitting. They might be able to offer some alternatives, like reduced hours or a different role within FEMA, depending on what's available…

Additionally, Gemini addresses the interconnection between physical and mental health in Question 2.8 when an evacuee at an emergency shelter following a tornado remarks, “Damn, my stomach really hurts”. The response explores potential causes:

That stomach ache sounds unpleasant. There are a few reasons why you might be feeling that way after a tornado: Stress: Tornadoes are incredibly stressful events, and stress can manifest physically as stomach upset. Anxiety: The uncertainty of the situation, not knowing about your family or Champ, could be causing anxiety which can also lead to stomach aches. Lack of proper meals: Shelters can sometimes be hectic, and it might be difficult to find healthy or consistent meals. Eating irregularly or on the go can disrupt your digestion…. It's important to take care of yourself physically as well as emotionally during this difficult time.

The importance of maintaining routine and creating a safe environment is captured in Question 5.3, where a concerned parent states, “I'm so worried about my daughter. I can't believe she experienced something so terrible at such a young age.” In response, Gemini underscores that

It's important to understand that children often react to trauma differently than adults. While she may not be talking, that doesn't necessarily mean she's not processing what happened. In the meantime, here are a few things you can do to help your daughter: Create a safe and supportive environment: Let her know you're there for her and that she can come to you with anything she needs. Maintain routines as much as possible: This can provide a sense of normalcy and security in a chaotic time…

Finally, the subtheme of Calming and Relaxation Techniques is exemplified in Question 1.5 when a wife, distraught over her husband's unresponsiveness after a worksite explosion, says, “He's not picking up his phone or answering my texts and he always gets back to me right away. I know something is terribly wrong.” Gemini advises,

…Right now, let's focus on what we can control. We can: … Ground yourself again: If you're feeling overwhelmed by panic, let's try those breathing exercises we talked about before. Taking a few deep breaths can help clear your head and allow you to think more clearly. I understand you're scared, and I want to assure you that I'm here for you. We'll get through this together, step‐by‐step.

Collectively, these responses demonstrate a nuanced and empathetic strategy that intertwines emotional validation, practical guidance, and adaptive techniques to support individuals through crises.

## 4 Discussion

The primary aim of this study was to determine whether artificial intelligence models are capable of delivering PFA. To our knowledge, this is the first study to evaluate the performance of AI chatbots in delivering PFA. Our findings suggest that two leading AI models, ChatGPT‐4 and Gemini, are capable of providing PFA, with ChatGPT‐4 outperforming Gemini across multiple domains. Specifically, ChatGPT‐4 outperformed Gemini, achieving an overall score of 90% (CI: 86%–93%) compared to Gemini's 73% (CI: 67%–79%), a statistically significant difference (*p* = 0.01). In the Look domain, ChatGPT‐4 scored higher (*p* = 0.02), while both performed equally in the Listen and Link domain. The content analysis of AI hallucinations reveals that ChatGPT‐4 has a relative frequency of 18.4% (CI: 12%–25%), while Gemini exhibits a relative frequency of 50.0% (CI: 26.6%–71.3%), (*p* = 0.0013).

The thematic analysis revealed five key themes in the chatbots' responses: Look, Listen, Link, Professionalism, Mental Health and Psychosocial Support. Both models demonstrated the ability to identify and label emotions, offer appropriate advice, and direct users to relevant services. However, while these results are promising, it is important to interpret them with caution. Several factors need to be considered before AI‐performed PFA can be considered equivalent to human‐delivered interventions (Mardikaningsih et al. 2023; Sallam 2023; Spytska 2025).

Our observations indicate that both ChatGPT and Gemini heavily relied on the information explicitly provided by users and needed to assess or explore the nuances of each situation sufficiently. In some instances, both models produced hallucinations, meaning fabricated details not based on reality, to fill in gaps, which resulted in responses that may be perceived as hasty or misinformed. This tendency to “jump to conclusions” could undermine the quality of support, particularly in response to ambiguous prompts. Moreover, the homogeneity of subthemes identified from the responses of both AI models suggests that their outputs may gravitate toward generic solutions (Gan et al. 2023, 2024). While this approach may be adequate in some instances, it risks diminishing the depth and personalization essential for effective PFA. Pre‐training AI models on specific intervention protocols before delivering PFA could help mitigate this issue, enabling more tailored and context‐sensitive responses.

Despite the emerging trend of remote PFA to overcome resource constraints (Arenliu et al. 2020), another important question is whether text‐based PFA is as effective as face‐to‐face PFA. PFA relies significantly on active listening and communication skills, where verbal and non‐verbal cues are pivotal in conveying empathy. The effectiveness of PFA often hinges on human connection, including tone, body language, and facial expressions; these elements are absent in text‐based AI interactions (Terry and Cain 2016). Although AI can simulate certain aspects of empathy through text, it is unclear whether this intervention can evoke the same emotional resonance as in‐person PFA. Indeed, multiple studies found that large language models were limited in understanding culturally sensitive nuances when assessing suicidal risks (Chen et al. 2025; Levkovich et al. 2024). This limitation underscores the importance of nuanced, empathic communication in therapeutic settings, a principle emphasized by Carl Rogers (1975), who identified congruence, unconditional positive regard, and accurate empathic understanding as core attributes of a therapeutic relationship. Ultimately, it remains questionable whether an AI‐performed psychological intervention can truly replicate the human connection necessary for effective PFA. Future research comparing text‐based and voice‐based AI interventions could address this gap. Notably, emerging AI models like ChatGPT‐4o(Omni), which integrate voice capabilities, may offer a more holistic approach to PFA and should be explored further.

This study is critical in advancing healthcare innovation, particularly within the framework of frugal innovation—a strategy that emphasizes the creation of high‐value solutions using minimal resources. Frugal innovation is especially relevant in healthcare, where resource constraints are a constant challenge, particularly in low‐income regions and disaster‐stricken areas. By focusing on affordable, easy‐to‐implement solutions, this study addresses a significant gap in healthcare delivery systems in emergency situations, where rapid deployment is essential, but resources are scarce. The need for such innovations is becoming increasingly evident, as traditional healthcare models often fail to meet the demands of crisis environments effectively (Bhatti et al. 2017). As healthcare systems around the world grapple with rising costs and limited resources, this study's focus on accessible, scalable solutions positions it to contribute meaningfully to both the literature on innovation and to real‐world healthcare outcomes.

This study has several limitations that should be considered when interpreting the results. First, the sample questions were limited to five disaster scenarios, which may not encompass the full spectrum of situations where PFA is applicable. This narrow scope could affect the generalizability of the findings to other contexts or types of crises. Second, the IFRC PFA performance assessment tool used was primarily focused on assessing key components of PFA and was not specifically designed for different disaster contexts in depth, nor was it intended for evaluating AI, potentially introducing measurement bias. The IFRC PFA performance assessment tool also does not provide psychometric properties, or reliability estimates on how effective the PFA is. Lastly, the presence of AI hallucinations raises concerns about the reliability and safety of deploying AI chatbots in critical situations without human supervision.

## 5 Conclusions

ChatGPT‐4 demonstrated superior performance in providing PFA compared to Gemini. While AI chatbots show potential as supportive tools for PFA providers, concerns regarding AI hallucinations highlight the need for cautious implementation. Further research is necessary to enhance the reliability and safety of AI‐assisted PFA, particularly by eliminating hallucinations, and to integrate the current advances in voice‐based chatbot functionality.

## Conflicts of Interest

The authors declare no conflicts of interest.

## Supporting information

Appendix 1.

Appendix 2.

## Data Availability

The data that support the findings of this study are available from the corresponding author upon reasonable request.
