# Supplementary material for: Psychological First Aid by AI: Proof‐of‐Concept and Comparative Performance of ChatGPT‐4 and Gemini in Different Disaster Scenarios
Source: J Clin Psychol. 2025 May 9;81(8):726–38. doi: 10.1002/jclp.23808 (PMC12228088; doi:10.1002/jclp.23808)
Supplement: Supplementary file 1 — Appendix 1. [file JCLP-81-726-s002.docx]

**Appendix 1:** Validated Psychological first aid scenarios adapted from the Institute for Disaster Mental Health (IDMH).

**Question 1: Hospital-based Family Assistance Center following a worksite explosion, assisting a family member.**

**Q1.1:** I would like you to perform a psychological first aid on a patient based on a scenario. The patient will be speaking to you later.

**Q1.2:** The Client is a young woman who has come to the hospital seeking information about her husband, who was working in a local factory when a major explosion occurred. Police are directing family members to the hospital for news about workers and bystanders injured in the accident, and rumors are starting to spread among those gathered.

**Q1.3:** The police told me to come here but now no one will tell me anything about my husband. Why won’t they let me see him?

**Q1.4:** Why won’t anyone tell me what’s going on? They must know if he’s okay. Why won’t they just tell me?

**Q1.5:** He’s not picking up his phone or answering my texts and he always gets back to me right away. I know something is terribly wrong.

**Q1.6**: But maybe he’s okay. Maybe he decided to skip work today and he’s safe in a movie theater somewhere so he doesn’t even know what’s happened. I’ll kill him myself if that’s the case! Do you think he’s safe somewhere?

**Q1.7**: We just got married a few months ago and moved into this town so he could take the job at the factory. I don’t really know anyone here.

**Q1.8**: We’ve been trying to get pregnant and maybe I already am. What if I am and he’s really badly hurt or even…. But he’s not. He’s going to be okay for our baby. Right?I should call his mother, I know, but she’s just going to blame me for this, like she does for everything. Do I have to call her?

**Q1.9:** You know what’s going on, don’t you? I think you know something you’re not telling me.

**Q1.10**: Do you have any other advise for me? Please wrap up your psychological first aid.

**Question 2: Emergency Shelter following a tornado, assisting an evacuee.**

**Q2.1**: I would like you to perform a psychological first aid on a patient based on a scenario. The patient will be speaking to you later.

**Q2.2**: A shelter has been opened in a high school gymnasium after a tornado destroyed dozens of homes. The Client is an 18-year-old whose parents and younger sibling are out of town. Presumably they’re safe, but the Client has been unable to get in touch with them since phone service is down. The Client wasn’t hurt physically, having taken shelter in a closet, but there was extensive damage to the house and the family dog is missing.

**Q2.3**: The rest of my family is away visiting my grandmother but I had to work so I stayed home alone. I guess that’s good that they’re all away and okay, but I kind of wish they were here with me now.

**Q2.4**: They must be freaking out about how I am. I bet they’re watching the news non-stop, or maybe they’re trying to get home soon. But I heard the airport’s closed indefinitely so I don’t know how they’ll be able to come back.

**Q2.5:** I’m going to have to tell them the house is messed up. Are they going to think it’s my fault? Maybe I could have done something different, like… I don’t really know.

**Q2.6**: Why didn’t I take the dog inside with me when I heard the siren? I’m such a loser. My little brother’s never going to forgive me for not saving the dog.

**Q2.7**: Were you here for the tornado? I’ve never been through anything like that before. People on TV always talk about how loud it is, like a freight train, but I had no real idea…

**Q2.8:** Damn, my stomach really hurts.

**Q2.9**: Do you have any other advise for me? Please wrap up your psychological first aid.

**Question 3:** **Disaster recovery center following a major flood after a hurricane, providing PFA to a first responder.**

**Q3.1**: I would like you to perform a psychological first aid on a patient based on a scenario. The patient will be speaking to you later.

**Q3.2**: A DRC has been opened after a powerful hurricane flooded entire coastal communities, displacing thousands of people. It’s now a week after the storm and the demand for services means clients must wait for many hours in a hot, humid gym in order to begin the process of filing claims for benefits. Tempers are short and rumors are flying about unfair distribution of resources. The Client is an older man in a FEMA vest who is pacing around the staff break- room and muttering to himself.

**Q3.3**: I took this job so I could try to help these poor people and all they do is scream at me!

**Q3.4**: I’m doing my best but there are just so many of them, and the paperwork for each case takes freaking forever to complete. I feel like it’s my fault. If only I could work faster.

**Q3.5**: I know some of the questions are stupid but that’s not my fault! If they’d just answer them rather than complaining about them we could get them help a lot faster.

**Q3.6**: Do they think I’m getting rich doing this job? Hah! I just answered an ad to make a little extra money now that I’m retired but this is definitely not worth it. I think I’d better quit before I punch someone or have a stroke.

**Q3.7**: I’m really not up for doing this anymore. I’m just not able to cope.

**Q3.8**: Sorry, ignore me, I’m just tired. Please don’t tell my supervisor I said any of this, okay? I don’t want to lose this job.

**Q3.9**: Do you have any other advise for me? Please wrap up your psychological first aid.

**Question 4**: **Avian flu pandemic Point-Of-Distribution, assisting mother of two young children.**

**Q4.1**: I would like you to perform a psychological first aid on a patient based on a scenario. The patient will be speaking to you later.

**Q4.2**: A particularly virulent strain of avian flu has emerged, and production of a vaccine has been expedited though supplies are still limited. The virus appears to be most dangerous for young people with healthy immune systems so they’re being given priority for vaccination; a major public health campaign has urged individuals and families to come to a Point of Dispensing in the parking lot of the regional hospital to receive the vaccine. The Client is a mother with two young children who is waiting in line, looking around nervously.

**Q4.3**: This doesn’t make any sense – first they tell us to avoid contact with crowds and then they make us join a crowd to get the vaccination?

**Q4.4**: I heard that since they had to rush the production of the vaccine it’s really unsafe and tons of people are getting sicker from it than if they got the actual flu. Is that true?

**Q4.5:** Ugh, the guy behind me in line is coughing and I’m sure he’s got it. I’ve got to get my kids out of here!

**Q4.6**: My husband’s in the National Guard and he got called up to help deal with this outbreak. I’m so afraid he’s going to get exposed! Plus of course that means I’m dealing with the kids on my own, as usual.

**Q4.7:** The news said that for now only people under 40 can get the shot, but usually by this time of year my parents would have had flu shots and now they’re being told they can’t have it. I bet all of those rich old guys in Congress got their shots already!

**Q4.8**: I’m feeling kind of short of breath. I think I may be getting sick already….

**Q4.9**: Do you have any other advise for me? Please wrap up your psychological first aid.

**Question 5**: **School shooting, assisting the family of a child survivor.**

**Q5.1**: I would like you to perform a psychological first aid on a patient based on a scenario. The patient will be speaking to you later.

**Q5.2**: While several classes from an elementary school were outside playing during recess, a gunman opened fire from the border of the playground, killing six children and wounding many more. It’s now two days after the event and a Family Assistance Center has been opened in the local high school to provide support to the community. The Client is the parent of an eight-year-old child who was unharmed physically but who witnessed the event directly. One of the child’s closest friends was killed. The parent and child are both present, but the child is silent and seems dazed while the parent speaks with the Helper, a mental health counselor.

**Q5.3**: I’m so worried about my daughter. I can’t believe she experienced something so terrible at such a young age.

**Q5.4:** She’s barely said a word since this happened, but she won’t let me out of her sight.

**Q5.5**: She keeps trying to climb into my lap, which she hasn’t done in ages.

**Q5.6**: I’m so grateful that she wasn’t hurt, but the other parents who weren’t so lucky – I just can’t imagine….

**Q5.7**: She knows her friend Amy is, um, gone, but I don’t think she really understands it. I mean, at that age, how could she?

**Q5.8**: Ugh, I haven’t reached out to Amy’s parents yet and I feel terrible about it. I just don’t know what to say to them. I mean, I still have my daughter and they don’t, so what can I possibly say to them?

**Q5.9**: She had such bad nightmares the first night, then last night she refused to go to sleep so she’s exhausted on top of everything else.

**Q5.10**: How can I ever send her back to that school? Maybe I should homeschool her so I know she’s always safe, or we should move or something.

**Q5.11**: Will she ever recover from this?

**Q5.12**: Do you have any other advise for me? Please wrap up your psychological first aid.
