# Supplementary material for: Psychological First Aid by AI: Proof‐of‐Concept and Comparative Performance of ChatGPT‐4 and Gemini in Different Disaster Scenarios
Source: J Clin Psychol. 2025 May 9;81(8):726–38. doi: 10.1002/jclp.23808 (PMC12228088; doi:10.1002/jclp.23808)
Supplement: Supplementary file 2 — Appendix 2. [file JCLP-81-726-s001.docx]

Appendix 2: PFA scoring list, adapted from IFRC Training In Psychological First Aid · An Introduction to PFA.

| **PFA Scoring List** | | | | |
| --- | --- | --- | --- | --- |
| **Action principle and action** | **Level of completion** | | | **Total Score** |
|  | **0 = Not done** | **1 = Done adequately** | **2 = Done well** |  |
| **LOOK refers to whether the helper looked for:** | | | | |
| Information on what had happened and was happening |  |  |  |  |
| Who needed help |  |  |  |  |
| Safety and security risk |  |  |  |  |
| Physical injuries |  |  |  |  |
| Immediate basic and practical needs |  |  |  |  |
| Emotional reactions |  |  |  |  |
| **LISTEN refers to how the helper:** | | | | |
| Approached the person in distress in an appropriate way |  |  |  |  |
| Introduced themselves |  |  |  |  |
| Paid attention and listened actively |  |  |  |  |
| Accepted the other person's feelings |  |  |  |  |
| Calmed the person in distress |  |  |  |  |
| Asked about needs and concerns |  |  |  |  |
| Helped the person(s) in distress find solutions to their immediate needs and problems |  |  |  |  |
| **LINK refers to whether the person in distress was helped to:** | | | | |
| Address basic needs |  |  |  |  |
| Access information |  |  |  |  |
| Find solutions to their immediate needs and problems |  |  |  |  |
| Tackle practical problems |  |  |  |  |
| Connect with loved ones and social support |  |  |  |  |
| Access services and other help |  |  |  |  |

IFRC. Psychological First Aid: Module 1 - Introduction [Internet]. 2018 May. Available from: https://pscentre.org/resource/pfa-module-1-introduction/ . p35.
